# The COVID-19 Pandemic: Is It A Wolf Consuming Fertility? 

**DOI:** 10.22074/ijfs.2020.6302

**Published:** 2020-07-15

**Authors:** Luigi Napolitano, Biagio Barone, Felice Crocetto, Marco Capece, Roberto La Rocca

**Affiliations:** Department of Neurosciences, Reproductive Sciences and Odontostomatology, School of Medicine, University of Naples "Federico II", Naples, Italy

Nowadays, male infertility is regarded as a global problem. Male infertility occurs due to abnormal sperm production or function that prevent the delivery and/or the quality of sperm. Infections, chronic illnesses, lifestyle choices and other factors can play a role in male infertility (1, 2).

Most of viral infections indeed, are able to affect both the reproductive tract tissue and the semen of humans and animals, impairing sperm parameters and DNA integrity by different pathogenetic mechanisms that are responsible for temporary or permanent infertility in males and females (1).

Considering the well-known evidence on relations between human immunodeficiency virus (HIV), human papilloma virus (HPV), cytomegalovirus (CMV), adenoviruses, parvovirus, mumps and fertility, the real question is “Can the SARS-CoV-2 pandemia possibly influence male patency?” Coronaviruses are enveloped, positive single-stranded large RNA viruses that infect both animals and humans (3).

The first example is the avian infectious bronchitis virus (AIBV), a coronavirus known to cause an acute respiratory infection in chickens. In addition to that, AIBV causes the formation of epididymal stones in roosters, with detrimental effects on sperm production and fertility. The presence of such calcium carbonate stones in the efferent duct of roosters causes the erosion of the epithelial lining, the reduction of testis weight and impairment of sperm production lowering the fertility for 35-40%.

Secondly, porcine reproductive and respiratory syndrome virus (PRRSV), a small enveloped RNA virus, shows a negative impact on fertility. In fact, the PRRSV primarily attacks pulmonary alveoli to further replicate in epithelial germ cells (mainly spermatids and spermatocytes) inducing apoptosis and increasing the number of immature sperm cells. The overall effect is a diminished quality of the semen. Moreover, this kind of replication permits sexual transmission of the virus that could be easily identified in ejaculate.

Thirdly, the blue tongue virus (BTV) - a double stranded RNA virus that affects rams – represent another example of viral infection-caused semen impairment. The BTV RNA was detected in 75-100% of semen samples obtained 25-57 days post clinical signs with notable impact on semen motility, concentration and vitality. Although very little is known regarding the pathogenic mechanisms, the increasing number of germ cell at different maturation stages and early signs of germinal epithelial regeneration suggest a previous severe degeneration and sloughing of germ cells.

Recent evidence reports that SARS coronavirus could also lead to a fertility damage, despite the absence of a direct mumps-like orchitis. It was hypothesized that SARS coronavirus may utilize the ACE-2 receptor expressed on testicular tissue. SARS patients’ testes displayed indeed a widespread germ cell destruction with thickened basement membrane and leukocyte infiltration, mainly macrophages, compatible with findings of previous animal studies (3). The presence of components of the renin angiotensin system (RAS) and specific receptors of angiotensin in the female and male reproductive tract supports the hypothesis that reproductive functions may be controlled by RAS (4). Angiotensin converting enzyme (ACE) is also involved in the regulation of blood pressure. One of the most active components of RAS system is angiotensin II (Ang II) that regulates cardiovascular and electrolyte homeostasis. Since Ang II was also found in seminal plasma, it might be able to act on mammalian spermatozoa where it is implicated in the maintenance of sperm function and fertility. Exposure of human spermatozoa to Ang II increases the percentage of motile spermatozoa and stimulates sperm linear velocity. Moreover, Ang II is involved in capacitation and acrosomal reaction. This ligand binds to two different receptors, AT1R and AT2R. These receptors were detected either in the male reproductive system and in spermatozoa. AT1R was detected in the tail and along the whole flagellum of spermatids and mature spermatozoa of humans and animals and found to be involved in capacitation and acrosome reaction. AT2R is mainly present in human semen at the equatorial/post acrosomal region of the head (5). The AT2R is located in the same region of μ-opioid receptor, pro-enkephalin, estrogen receptor-α, and γ-aminobutyric acid A receptor. Such proteins are all involved in cell transduction and sperm motility. It was suggested that AT2R may be involved in the control of sperm motility as well (6). Coronaviruses isolated from bats since 2005 showed a particular propension to cross species barriers, infecting the lung cells of mammals utilizing the ACE- 2 receptors and exerting a potential zoonotic-reverse zoonotic cycle that allow the virus to maintain viral population in multiple hosts (7). The reported different spillover episodes, the well-established reproductive problems related to coronaviruses in mammals and birds and finally the evidence regarding the presence of ACE-2 receptors in human genital tract does not let us excluding potential reproductive issues in humans. Particular attention should be given to asymptomatic patients who are often the major carriers of the Covid-19 infection (8). It is also necessary to identify all potential clinical presentations and the possible, long-term consequences of Covid-19 -infection. According to the literature data, a possible reproductive system localization and, particularly spermatozoa localization with possible implications for male fertility, cannot be excluded. Further studies are needed to better define the physiopathology and clinical implications of respiratory virus infections on male fertility.
